# Drug-Loaded Nanoparticle Systems And Adult Stem Cells: A Potential Marriage For The Treatment Of Malignant Glioma?

**DOI:** 10.18632/oncotarget.937

**Published:** 2013-03-26

**Authors:** Brenda Auffinger, Ramin Morshed, Alex Tobias, Yu Cheng, Atique U Ahmed, Maciej S Lesniak

**Affiliations:** ^1^ The Brain Tumor Center, The University of Chicago, Chicago, Illinois, USA

**Keywords:** Nanoparticle, drug delivery systems, stem cell carriers, malignant glioma, brain cancer, targeted delivery

## Abstract

Despite all recent advances in malignant glioma research, only modest progress has been achieved in improving patient prognosis and quality of life. Such a clinical scenario underscores the importance of investing in new therapeutic approaches that, when combined with conventional therapies, are able to effectively eradicate glioma infiltration and target distant tumor foci. Nanoparticle-loaded delivery systems have recently arisen as an exciting alternative to improve targeted anti-glioma drug delivery. As drug carriers, they are able to efficiently protect the therapeutic agent and allow for sustained drug release. In addition, their surface can be easily manipulated with the addition of special ligands, which are responsible for enhancing tumor-specific nanoparticle permeability. However, their inefficient intratumoral distribution and failure to target disseminated tumor burden still pose a big challenge for their implementation as a therapeutic option in the clinical setting. Stem cell-based delivery of drug-loaded nanoparticles offers an interesting option to overcome such issues. Their ability to incorporate nanoparticles and migrate throughout interstitial barriers, together with their inherent tumor-tropic properties and synergistic anti-tumor effects make these stem cell carriers a good fit for such combined therapy. In this review, we will describe the main nanoparticle delivery systems that are presently available in preclinical and clinical studies. We will discuss their mechanisms of targeting, current delivery methods, attractive features and pitfalls. We will also debate the potential applications of stem cell carriers loaded with therapeutic nanoparticles in anticancer therapy and why such an attractive combined approach has not yet reached clinical trials.

## 1 INTRODUCTION

Glioblastoma multiforme, the most common primary brain tumor in adults, is characterized by a highly invasive phenotype and invariable recurrence. Despite aggressive standard therapy, which consists of surgical resection, followed by chemo- and radiotherapy, the median survival remains only 14.6 months [1, 2]. Several problems have been held responsible for such a dismal prognosis. First, due to diffuse malignant infiltration into white matter tracts, complete surgical extirpation of the tumor is considered impossible. Second, most effective drugs available in the market are not able to cross the extremely selective blood-brain barrier (BBB) that surrounds the central nervous system (CNS). In addition, the few chemotherapeutic agents that are able to reach the brain post-systemic administration usually do not get to the tumor site at a concentration high enough to kill infiltrative tumor cells without damaging non-neoplastic tissues [3]. Last, cancer stem cells, which have been shown to play a major role in therapeutic relapse and tumor recurrence, have proven to be resistant to the current standard therapies [4].

Several efforts have been made to overcome these obstacles. Among them, the development of nanoparticle delivery systems has offered a new hope in obtaining efficient and effective therapeutic distribution. Most of the newly engineered nanoparticles are able to cross the BBB and accumulate within the tumor. Their surface can be functionalized in order to enhance a targeted delivery to neoplastic tissues [5]. These nanocarriers can also be loaded with a wide range of diagnostic and therapeutic agents, allowing for effective uptake across the BBB of previously non-diffusible chemotherapeutic drugs [6, 7]. Moreover, their versatile structure allows for the loading of a high number of therapeutic agents per nanocarrier, which, together with a targeted delivery, increases the amount of drugs that accumulate at the tumor site, and decreases toxicity to normal tissues. As a result of such superior delivery, this new therapeutic approach has proven to target both chemoresistant cancer stem cells and rapidly dividing malignant cells [8].

Although powerful tools for both diagnosis and treatment of brain tumors, nanoparticle delivery systems alone present several drawbacks that hamper complete tumor eradication. First, depending on the size of the carrier, cells from the mononuclear phagocyte system, such as macrophages and lymphocytes, can easily engulf them, which decreases the amount of systemically administered nanoparticles that effectively reach the tumor area [9, 10]. Second, upon reaching glioma tissues, nanoparticle systems may experience uneven intratumoral distribution due to entrapment in the extracellular matrix space surrounding neoplastic cells or within intratumoral necrotic pockets. Third, although these carriers are able to reach the tumor site, they are mostly unable to effectively target infiltrative areas.

The use of stem cell carriers loaded with nanoparticle delivery systems has recently been presented as a solution to overcome the above limitations. They have proven to be effective delivery vehicles for many therapeutic systems, including nanoparticles [11-14]. They can be loaded with a fair amount of these nanocarriers, while keeping their inherent tumor-tropic properties. In addition, they are able to migrate within glioma tissue, providing increased intratumoral distribution of the therapeutic payload and targeted migration to infiltrative tumor areas [11]. They were shown to protect these therapeutic agents from the host immunosurveillance, decreasing their unrestricted uptake by mononuclear cells. Furthermore, they possess intrinsic immunosuppressive properties, which have been shown to decrease tumor inflammation and improve therapeutic index [15]. In this review, we will describe the main nanoparticle delivery systems that are presently available in preclinical and clinical studies. We will discuss their mechanisms of targeting, current delivery methods, attractive features and pitfalls. We will also debate the potential applications of stem cell carriers loaded with therapeutic nanoparticles in anticancer therapy and why such an attractive combined approach has not yet reached clinical trials.

## 2 Nanoplatforms

Nanoparticle platforms are defined by their physicochemical structure that gives them their unique and multifunctional properties. At their foundation, nanoparticles are materials that range from one to a hundred nanometers in at least one dimension, have a large surface to volume ratio, and can, to some extent, cross the BBB [16-18]. Beyond these commonalities, a huge diversity of nanoparticle systems has been developed to possess different shapes, sizes, chemical properties, and biofunctional compositions that render them unique from one another [19]. In the following section, we will describe the most widely studied organic and inorganic nanoparticles in the context of glioma therapy [17].

### 2.1 Liposomes

Liposomes are spherical polymeric vesicles made up of a lipid bilayer, and range in size from 100 nm to 5 μm in diameter [20]. Liposomes have the ability to carry hydrophilic and hydrophobic molecules [21]. One major advantage of liposomes is that they are easily manipulated during their synthesis process [16]. By taking advantage of this, many liposomes have been constructed to be temperature- or pH--sensitive, allowing for regulated release of their contents [22]. Moreover, due to their physiochemical characteristics, liposomes have long blood circulation times and favorable diffusion properties [23].

### 2.2 Micelles

Micelles range in size from 20 nm to 200 nm in diameter, and are formulated from lipids or other amphiphilic molecules. These nanostructures have a hydrophobic core with a hydrophilic exterior [21], which has the advantage of rendering insoluble therapeutic agents soluble in biologic environments [24]. The outer shell of a micelle provides added protection over freely administered therapeutics, thus increasing the amount delivered to the intended target [25]. Furthermore, micelles can be structurally and chemically modified to respond to various environmental stimuli (i.e. pH, temperature) therefore regimenting therapeutic release [26].

### 2.3 Polymeric nanoparticles

Polymeric nanoparticles are assembled from either natural or synthetic polymers and may be the best nanoparticles for long-term therapeutic delivery [16]. These nanocarriers have a hydrophobic core with a large loading capacity, and a hydrophilic shell that provides stability and protection [27]. They have been designed to encapsulate both hydrophobic and hydrophilic molecules, as well as macromolecules such as proteins and nucleic acids [28]. Polymers have demonstrated low levels of toxicity, and are naturally metabolized and secreted from the body [16]. Lastly, break-down and release of therapeutics can be regulated in a controlled manner by altering the physiochemical properties of the nanoparticle, such as its molecular weight, dispersity index, hydrophobicity, and crystallinity [16].

### 2.4 Dendrimers

Dendrimers are polymer-based nanoparticles that range in size from 5-10 nm in diameter and contain extensive branching arms and multivalent functional groups. The tree-like structure of dendrimers allows for multifunctional properties. Dendrimers are capable of enclosing therapeutics within their structure, while anchoring imaging agents and targeting molecules to its periphery. Additionally, controlled degradation of dendrimers and release of therapeutics can be achieved through the thoughtful choice of their polymer chain chemistry [29].

### 2.5 Iron oxide nanoparticles

Iron oxide nanoparticles are magnetic nanoparticles that have a diameter of 10-100 nm [21]. The chemical properties of iron oxide nanoparticle surface modifications, such as inorganic silica or natural polymers, are required to stabilize the nanoparticle and are also used to attach ligands for targeting [30]. The relaxivity of the core of iron oxide nanoparticles makes them particularly potent magnetic resonance contrast agents [31]. A recent study has shown that the use of superparamagnetic iron oxide nanoparticles (SPIONs) for magnetic resonance imaging (MRI) as compared with conventional MRI contrast agents, increased both the diagnostic sensitivity and specificity in the detection of metastatic tumors [17]. Magnetic hyperthermia (MHT) is a therapeutic application of iron oxide nanoparticles that takes advantage of their magnetic properties [32].

### 2.6 Gold nanoparticles

Gold nanoparticles consist of self-assembled gold atoms that range in size from 1-150 nm in diameter [33]. The synthesis of gold nanoparticles results in high reaction yields with a consistent nanoparticle size, shape, and mass [34]. Gold nanoparticles are thought to be biologically inert and therefore have a low toxicity and high biocompatibility [35]. Another advantageous characteristic of gold nanoparticles is their strong optical property, due to surface plasmon resonance, that is detectable in the visible region of the light spectrum [36]. Lastly, because of the surface chemistry of gold nanoparticles functional diversity can be achieved with relative simplicity [37].

## 3 Nanoparticle delivery in glioma therapy

The inherent nature of glioma presents several distinct challenges for the successful delivery of therapeutics. Primary targeting is the first major obstacle and involves delivering nanoparticles past the highly discriminating BBB; a physiological barrier of the brain that limits unrestricted diffusional movement of molecules into and out of the brain [38]. The second challenge is specifically targeting glioma cells such as the aggressively infiltrative cells that have left the primary tumor site and spread throughout the brain. These invasive cells eventually give rise to recurrent disease [39]. This is referred to as secondary targeting. The following section will review the ways nanoparticle systems have aimed to overcome these challenges, and outlines the major roadblocks that still remain to be resolved.

### 3.1 Primary targeting - the brain

Nanoparticles have been delivered to malignant gliomas both systemically and locally. Systemic delivery of nanoparticles via intravenous injection requires efficient transport through the systemic circulation and across the BBB in order to deliver therapeutics at an optimal distribution and therapeutic concentration into the CNS [23]. Local delivery circumvents the BBB and relies on the diffusion of nanoparticles directly into the brain parenchyma [40].

#### Systemic Delivery

Intravenous injection is the most widely used method to deliver nanoparticles to the brain because of its non-invasive nature [41]. Although intravenous administration presents a viable option, nanoparticle delivery is hindered because of clearance by the mononuclear phagocyte system [42]. To circumvent this, nanoparticles smaller than 100 nm with amphipathic poly (ethylene glycol) (PEG) surface modifications have been shown to mitigate macrophage recognition and increase blood circulation half-life. However, the lack of an inclusive study of this effect across multiple cell lines and animal models has created conflicting results [42, 43]. Furthermore, to address the issue surrounding the BBB, it has been reported that BBB penetrance occurs with nanoparticles in the 15-100 nm range but optimal passage occurs with nanoparticles ≤15 nm in size. Therefore, particle size poses a clear limitation on what type of nanocarriers can be utilized for brain tumor diagnosis and treatment [44-46].

Beyond particle size, nanoparticles have been modified with cationic bovine serum albumin (CBSA), aclarubicin-loaded CBSA, or polysobate 80 surfactant, and these modifications have resulted in more robust levels of nanoparticle accumulation within the brain [7, 47, 48]. Also, modifying nanoparticles with lipophilic additives reduces their surface charge therefore binding apolipoprotein E (ApoE), which can enhance BBB uptake [49, 50]. Although these modifications help to increase the uptake of nanoparticles into the brain, local injection methods have generated a greater accumulation of nanoparticles in this organ [41].

#### Local Delivery

Local or direct bolus injection of nanoparticles is an alternative delivery method that bypasses the systemic circulation and BBB altogether [41]. A major disadvantage of this delivery technique stems from the tightly packed cells in the brain matter that results in low diffusion coefficients and causes sluggish diffusion and backflow of the injected nanoparticles [41, 51]. Additionally, high intracellular fluid pressure in gliomas further restricts local delivery [52, 53]. In order to correct these issues, a technique called convection-enhanced delivery (CED) has been applied with local injection of nanoparticles to create a pressure-driven gradient that drives nanoparticle diffusion [54, 55]. CED has shown encouraging results in experimental animal models [56]. However, issues such as the limited amount of time CED can be applied [57], variable distribution, as well as safety concerns, render CED as a questionable mode of delivery of nanoparticles to glioma tissues [58, 59].

### 3.2 Secondary targeting - the glioma cell

The ability to specifically target glioma cells either within the primary tumor or cells that have diffused deeply throughout the normal brain tissue is a complex challenge. One way nanoparticles may overcome this obstacle is through passive targeting. Passive targeting occurs naturally through the enhanced permeability and retention effect (EPR). The second tactic is active targeting which involves functionalizing the surface of nanoparticles with glioma-specific targeting moieties.

#### Passive targeting - enhanced permeability and retention effect

The enhanced permeability and retention effect (EPR) is a phenomenon involving solid tumors where their porous vasculature and secretion of elevated levels of vascular permeability factors create an environment that promotes tumor growth (i.e. necessary levels of oxygen and nutrients) [60]. Since the onset of this discovery, cancer therapies have exploited the distinct tumor microenvironment to deliver therapeutics to cancer tissue [61]. Within the context of brain tumors, passive targeting is especially significant as glioma vasculature is considerably hypervascularized, leaky, and deficient of a proper lymphatic drainage system. This creates an ideal environment for the uptake and retention of nanoparticles in tumor tissues, while sparing healthy brain cells [62]. It has been shown that passive targeting can be enhanced by localizing supermagnetic nanoparticles within glioma through an externally applied magnetic field [63]. In one study using this approach, magnetic paclitaxel nanoparticles significantly increased the survival of glioma-bearing rats when compared with free paclitaxel [64]. Regardless, the efficacy of EPR-based nanoparticle delivery for glioma therapy is still under question because of glioma's pathophysiological heterogeneity. It has been well reported that the core of gliomas consists of hypovascularized or necrotic tissue. Therefore, these central areas of the tumor do not exhibit an active EPR effect [65]. Moreover, it is thought that the existence of a “hypoxic niche” supports the small subpopulation of tumor stem cells that are responsible for glioma growth, progression, and disease recurrence [66, 67]. The access of nanoparticles to the hypoxic core may therefore be crucial for therapeutic impact.

#### Active Targeting - functionalizing the surface of nanoparticles

Active targeting involves functionalizing the surface of nanoparticles by attaching ligands or antibodies specific to glioma on their surface [68]. Several nanoparticles utilizing active targeting have been developed for glioma treatment both *in vitro* and *in vivo*. In one *in vitro* study, an antibody specific to interleukin-13 receptor alpha 2 (IL-13Rα2) (a receptor up-regulated in glioma cells) was attached to the surface of nanoparticles and demonstrated tumor specificity and toxicity in U373 and U87 glioma cell lines [69]. Additionally, Madhankumar et al. used an IL-13 conjugated nanoliposome to target U251 glioma cells *in vitro* and revealed increased internalization compared to their unconjugated counterpart. In an intracranial animal model using human U87 cells, this functionalized nanoparticle system resulted in a 5-fold reduction in intracranial tumor volume as well as significantly extended survival compared to animals receiving unconjugated liposomes [70]. In another study, carbon nanotubes were functionalized with the monoclonal antibody CD133^+^, a marker thought to be specific for glioma stem cells [71]. This study revealed the ability to selectively target and destroy CD133^+^ glioma stem cells and not CD133^−^ cells, as well as thwart their tumorigenic and self-renewal capacity *in vitro*. Furthermore, similar antitumoral effects were exhibited in a xenograft nude mouse model [72]. Another moiety used in active nanoparticle targeting of glioma is the tumor-associated antigen (TAA) epidermal growth factor receptor vIII (EGFRvIII). It has been shown that elevated levels of EGFR protein and EGFR amplification are present in 40-60% of glioblastomas [73]. The results of one study using an EGFRvIII antibody-conjugated iron oxide nanoparticle exhibited survival efficacy and served as a suitable targeted therapy towards infiltrative glioblastoma [74]. Beyond antibodies and antigens, ligands have also been used for active targeting of nanoparticles in glioma therapy. For example, a polymeric nanoparticle introducing a plasmid encoding proapoptotic Apo2 ligand tumor necrosis factor-related apoptosis-inducing ligand (Apo2L/TRAIL) repressed tumor growth and lengthened survival in a C6 murine glioma model [75]. Lastly, Kang et al. have developed a polymer based nanoparticle modified with a transferrin (Tf) ligand that precisely binds to the Tf receptor which is upregulated on the surface of proliferating glioma cells [76]. This nanoparticle system revealed improved cytotoxicity and extended survival of C6 tumor-bearing rats by an average of 88.37% when treated at an early stage of disease progression [77]. Despite the progress made with active nanoparticle targeting, glioma's prodigious intra- and inter-tumoral heterogeneity as well as the aggressiveness of infiltrating tumor cells continue to challenge the success of nanoparticle systems.

Even after the implementation of CED as an attempt to overcome the shortcomings of the local delivery, the use of nanoplatforms alone in anti-cancer therapy still faces some important challenges such as uneven intratumoral distribution and ineffective targeting of distant tumor foci. One possible way to overcome these problems is the use of cell carriers as vehicles for drug-loaded nanoparticle delivery to the targeted tumor site. Amongst other cell carriers, neural and mesenchymal stem cells (NSCs and MSC, respectively) are especially attractive for this application because they have proven to be permissive to nanoparticle incorporation, immunoprotective, and tumor tropic. These specific stem cell properties make delivery of nanoparticle payloads to targeted tumor areas in an optimal concentration a realistic possibility. Moreover, their unique oncotropic property allows them to target distant tumor foci, and potentially decrease the levels of tumor recurrence. As a result, stem cell carriers seem to be more suitable as delivery vehicles for anticancer therapy when compared to other options. In the following sections, we will survey stem cells as tumor-specific carriers of drug-loaded nanoparticles, as well as address their synergistic effect in anti-glioma therapy and the main limitations related to such an approach.

## 4 Stem cells as carriers of nanoparticle-delivery systems

### 4.1 Neural stem cells

NSCs are defined by their ability to self-renew and differentiate into neurons, astrocyte, and oligodendrocytes, the three major cell types in the CNS. In the adult brain, they are located within the subventricular zone and the dentate gyrus of the hippocampus. These cells demonstrate homing behavior towards glioma cells both *in vitro* and *in vivo* [11, 78, 79]. NSCs have already begun to make their way into clinical trials in the field of regenerative medicine for diseases such as ALS, Parkinson's disease, and stroke [80]. Information about their fate and migratory abilities gained from these studies will no doubt be important for further advances in delivery of anti-cancer agents using stem cells. The Food and Drug Administration (FDA) has also recently approved the HB1.F3.CD NSC line for their evaluation in clinical trials for the treatment of malignant gliomas. The goal of the study is to evaluate the safety of NSCs expressing the suicide gene cytosine deaminase (CD), which can convert the pro-drug 5-Fluorocytosine to the active drug 5-Fluorocytosine (NCT01172964). Other therapeutic agents, including oncolytic viruses, have already been loaded in NSCs to target gliomas in animal models, and further clinical studies in patients with brain cancer may be on the horizon [11, 81]. As these clinical studies progress, more information about the safety and challenges of using NSCs for therapeutics delivery will become evident.

### 4.2 Mesenchymal stem cells

MSCs are multipotent cells typically found in the bone marrow [82] that can give rise to a variety of cell types including osteoblasts, chondrocytes, and adipocytes [83]. They have been isolated from many locations including bone marrow, peripheral blood, lung, adipose tissue, as well as a variety of other sites [84-88]. These stem cells have also exhibited tumor-homing capabilities both *in vitro* and *in vivo* [89-92], suggesting their potential as therapeutic carriers. Many studies have loaded non-nanoparticle therapeutic agents into MSCs to target malignant gliomas [89-91, 93, 94]. For example, Kosaka et al. reported that MSCs expressing CD and concurrent 5-fluorocytosine administration could improve the survival of rats bearing 9L gliomas [94]. Another report by Ryu et al. demonstrated that MSCs loaded with herpes simplex virus type I thymidine kinase could increase the survival of glioma-bearing mice [93]. There are also a number of clinical trials that have already used MSCs for therapeutic delivery or for regenerative purposes, including chronic ischemic stroke and ALS [80] (NCT01051882; NCT01287936). Even though they are not aimed at treating malignant gliomas, lessons learned from these current studies will provide critical insight into the efficacy, fate and safety of these cell types.

### 4.3 Tumortropic and immunosuppressive properties of stem cell carriers

In the context of malignant gliomas, stem cells may provide a more targeted delivery method over other previously described cell carriers, such as macrophages [95], endothelial cells [96], cytokine-induced killer (CIK) cells [97], dendritic cells [98], monocytes [96] and T-cells [98, 99]. MSCs and NSCs have demonstrated homing behavior towards glioma cells both *in vitro* and *in vivo* [11, 78, 79, 89-92]. Not only do these cells migrate and distribute themselves within the tumor bed after a short time period, NSCs and MSCs also appeared to trail invading cancer cells [78]. The mechanisms of this migratory behavior are still unclear but have been shown to involve signaling pathways including the SDF-1 (stromal cell-derived factor-1)/CXCR4 (C-X-C chemokine receptor type 4) system [100] as well as other signaling pathways involving uPA/uPAR (urokinase receptor), VEGF/VEGFR2 (vascular endothelial cell growth factor receptor 2), and c-Met (pro-oncogene) receptors [101]. Because of this targeted migratory ability, it is thought that stem cells might act as carriers of therapeutic agents, including nanoparticles. Another important characteristic of both MSCs and NSCs that makes them attractive cell carriers is their intrinsic immunosuppressive properties. Such immunosuppression can be explained by four main mechanisms: promotion of apoptosis of type 1 T-helper cells, inhibition of pro-inflammatory cytokines, expression of MHC-I (major histocompatibility class I) and absence of MHC-II antigens [102]. The last two mechanisms, respectively, are responsible for the protection of stem cell carriers from natural killer (NK) cells, and allow for immune evasion from CD4^+^ lymphocytes [15]. Therefore, both the tumortropic and immunosuppressive properties of stem cell carriers make them excellent choices for targeted anti-glioma therapy.

### 4.4 Incorporation of nanoparticles into the cell carrier: trafficking and storage

**Figure 1:**
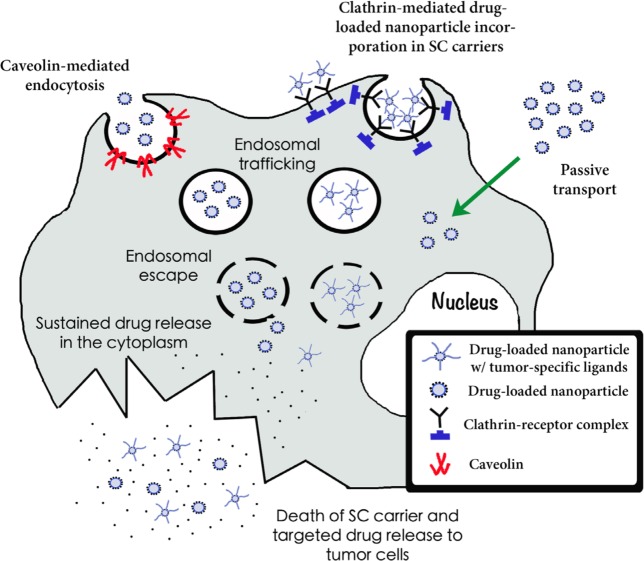
Incorporation, trafficking, endosomal escape and sustained drug release of nanoplatforms into stem cell carriers Three mechanisms of nanoparticle incorporation into cell carriers are depicted here: caveolin-mediated endocytosis, clathrin-mediated endocytosis and passive transport. Drug-loaded nanocarriers coated with cationic charges or tumor-specific ligands are incorporated into stem cells. By different mechanisms (see text) endo-lysosomal escape takes place. Drug-loaded nanoparticles then accumulate in the cytosol. Nanoparticle accumulation and sustained drug release in the cytoplasm of the cell carrier leads to membrane disruption and targeted drug release to tumor cells.

Nanoparticle uptake by stem cell carriers can be variable depending on several factors including the type of particle, size, and surface charge. Furthermore, several uptake mechanisms may be at play simultaneously. Some possible uptake mechanisms include passive Brownian diffusion into the cell, protein-mediated transport, clathrin-mediated, and caveolin-mediated endocytosis. Nativo et al. demonstrated that surface modifications of gold nanoparticles using cell penetrating peptides could affect their intracellular distribution, suggesting changes in uptake mechanisms [103]. Silica nanoparticles, however, were shown to interact with the lipid membrane of large unilamellar liposomes, inducing transmigration [104]. Mesoporous silica and polystyrene nanoparticles have been assimilated by ovarian cancer cells via caveolin-mediated endocytosis [105]. Once inside the cells, drug-loaded nanoparticles are transported to the endo-lysosomal system, where they are destroyed. Due to secondary changes in their surface charge, which leads to an interaction between nanoparticles and lysosomal membrane, polylactide (PLA) and polyglycolide (PLGA) polymer nanoplatforms are able to disrupt the lysosomal membrane and escape into the cytoplasm, where they accumulate [106]. Lipid nanoparticles, in turn, suffer lysosomal escape through direct interaction of their outmost coat, the hydroxystearate of poly(ethylene glycol) (HS-PEG), with the membrane of the lysosome [107]. Nanoparticle accumulation and sustained drug release in the cytoplasm of the cell carrier leads to membrane disruption and targeted drug release in tumor cells (Figure 1). The study of the means by which nanocarriers are assimilated and trafficked into stem cells is still in its infancy. A deep understanding of the uptake mechanism of most nanoparticle systems is still lacking, and if more analyses are conducted in this arena, it might provide insight on how to optimize uptake and distribution of these particles.

## 5 Delivery methods of stem cells to the brain

### 5.1 Intracranial injection

**Figure 2:**
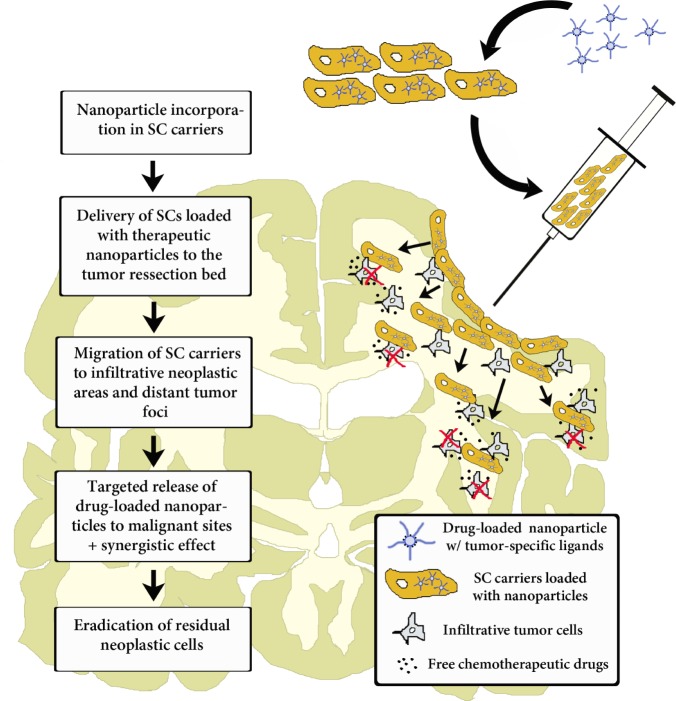
Intraoperative transplantation of stem cells carrying drug-loaded nanoparticles into the human brain post-tumor resection Chronological order of the events that take place post-surgical transplantation. Here, stem cell carriers' tumor-tropic migration results in a targeted drug release in infiltrative tumor zones. Modified cell carriers contribute to the local toxic effects caused by the drug-loaded system in neoplastic areas. Stem cells' immunosuppressive properties hide loaded nanocarriers from the host-immune system and facilitate targeted anti-glioma therapy.

Most of the current clinical trials using NSCs involve direct perioperative intracranial injections [80, 108] (NSC01172964; NCT01151124; NCT01005004) (Figure 2). Patients with Batten disease, for example, received multiple subcortical and intraventricular injections of NSCs, and the number of cells injected was found to be well tolerated with the use of immunosuppressant therapy. However, if this injection method is to be used effectively for brain tumor diagnosis and therapy, stem cells loaded with therapeutic agents will need to demonstrate increased efficacy over intratumoral injections of the therapeutic agent alone. It is possible that the ability of these cells to distribute themselves within the tumor and track down invading cells may lead to enhanced efficacy.

### 5.2 Intranasal injection

Intranasal delivery of stem cells has also been demonstrated as a less invasive method of transporting these cells into the CNS. Danielyan et al. delivered MSCs to the brains of 6-hydroxydopamine (6-OHDA) unilaterally lesioned rats, a model of Parkinson Disease [109]. The authors noted distribution of MSCs throughout the CNS and increased tyrosine hydroxylase levels in the lesioned ipsilateral striatum and substantia nigra, leading to behavior improvements. Importantly, 24% of the injected MSCs survived in these rats for at least 4.5 months, suggesting long-term integration and survival of injected cells. Velthoven et al. used intranasal delivery of MSCs in the context of CNS ischemia [110]. It was noted that these MSCs could reduce lesion size and induce functional recovery in a cerebral hypoxia-ischemia mouse model. Intranasal injection of stem cell carriers has also been described in the context of malignant gliomas. In a recent report, Reitz et al. analyzed the tumortropic and migratory pathways of human- and murine-derived NSCs after intranasal administration in mice bearing intracerebral glioblastomas. Intranasally delivered NSCs displayed a rapid and targeted tumor tropism, with most of the cells reaching the glioma site within 6 hours after injection. Histological sections revealed the olfactory bulb and the microvasculature of the nasal mucosa as the two main pathways of stem cell migration [111]. Taken together, these studies provide key insight into an effective and less invasive method for introducing stem cells into the CNS.

### 5.3 Intravenous injection

Aboody et al. first demonstrated that intravenously injected NSCs could migrate to brain tumors in a nude mouse xenograft glioma model. However, the authors did mention that such a delivery method resulted in low migration efficiency [78]. Intravenous-injected MSCs have already been used in clinical trials for other neurological diseases such as stroke. Bang et al. described a randomized clinical trial in which stroke patients were injected intravenously with MSCs, and no adverse events were noted in these 5 patients by 5-years [112, 113]. Honmou et al. reported 12 stroke patients who were injected intravenously with bone marrow-derived MSCs [114]. There was no tumor or abnormal cell growth in these patients over 1 year. Nevertheless, this intravenous delivery method brings up some obvious concerns surrounding the distribution of these cells. More work must be done to study the biodistribution and fate of these intravenously injected cells.

## 6 Currently available methods incorporating nanoparticles and stem cell carriers

### 6.1 Gene therapy

MSCs have demonstrated the ability to express transgenes efficiently without any alteration of their tumortropic or immunosuppressive properties [115]. Such competent expression may offer an anti-cancer therapy where the product of a gene within a stem cell could be used to target surrounding glioma tissues. Gene therapy usually relies on the use of viral vectors as transporters to deliver the desired gene to cell carriers. However, this method faces many limitations, including infection-related cell damage and immune response issues [116]. Nanoparticles bound with DNA avoid many of these problems and have shown promise for gene delivery into stem cell carriers. Park et al. developed polyethylenimine (PEI)-DNA coated silica nanoparticles for gene delivery into hMSCs [117]. The authors reported that 75% of hMSCs showed uptake of this vehicle. In another study, the same group demonstrated that PEI polyplexed with transcription factor SOX 5, 6, and 9 coated onto PLGA nanoparticles led to a significant increase in chondrogenesis of hMSCs *in vitro* [118]. Yang et al. delivered the hVEGF gene to hMSCs using biodegradable poly beta-amino ester nanoparticles, leading to enhanced VEGF production in these cells. This led to enhanced angiogenesis and limb salvage [119]. Although such reports were not specific to glioma therapy, the concept of nanoparticle-mediated gene delivery to stem cells may be of value if anti-glioma genes can be incorporated onto nanoparticle transporters. The goal of such transporters would be to enhance gene uptake by stem cells without the limitations found in viral gene delivery, a method already demonstrated in the treatment of glioma [11, 81].

### 6.2 Labeling of stem cells to track migration

Labeling of stem cells with nanoparticles to track their migration is one of the best-established combinations of nanoparticles and stem cells in the field of medical imaging. Magnetic iron oxide nanoparticles such as SPIOs have been commonly used to enhance imaging in addition to other non-iron particles including manganese oxide nanoparticles and gadolinium chelates attached to the surface of nanoparticles [120]. SPIOs have also been used to track the migration of stem cells and demonstrate the migratory path towards tumors [92, 120]. Wu et al. demonstrated that while initially MSCs were scattered throughout the tumor at early stages after injection, after 14 days the MSCs were found at the border between tumor and normal parenchyma [92]. Thus, such cells loaded with SPIOs may offer a more efficient way of delineating the boundaries of the tumor using MRI. Flexman et al. provided a quantitative analysis of the migration of NSCs loaded with ferumoxide (SPIO + dextran) and found that NSCs typically migrate at a speed of 50-70 μm/day depending on the brain region [121]. The study also claimed to be able to distinguish cell migration from clearance or degradation of cell debris and free tracer. It has also been demonstrated that nanoparticle systems such as ferumoxide do not disrupt the migratory ability of MSCs towards glioma cells [122]. Concerning the NSC line newly approved for clinical trials, Thu et al. demonstrated that loading with ferumoxide-protamine sulfate complex nanoparticles did not change the migration properties of HB1.F3.CD cells. These results support the use of nanoparticles to improve our understanding of the distribution of stem cells systemically and throughout the brain and help delineate the migratory mechanism of cell carriers to tumor burdens, and support their use in future clinical studies [123].

### 6.3 Delivery of toxic agents

One of the major challenges of the stem cell-nanoparticle partnership is developing ways to deliver toxic compounds to areas of the tumor in a specific manner while preserving carrier migration. Li et al. demonstrated that silica nanorattle-doxorubicin particles could be anchored to MSCs in a system called “nanoparticulate patches” [124]. Loaded cells were able to migrate towards U251 cancer cells both *in vitro* and *in vivo*. Furthermore, this system demonstrated more widespread apoptosis at 7 days post-intratumoral injection of silica nanoparticle-doxorubicin loaded MSCs when compared to injections of free doxorubicin.

Roger et al. demonstrated that marrow-isolated adult multilineage inducible cells (MIAMI cells) containing ferrociphenol could induce cytotoxicity in U87MG glioma cells *in vitro* via a transwell system assay [125]. Tumors treated with this delivery system showed decreased growth rates *in vivo*. Nevertheless, the results were not very robust and survival improvements of the animals were not discussed. In addition, the authors did not mention toxicity improvements of the stem cell-nanoparticle combination therapy over treatment with stem cells alone. In a report from Rachakatla et al., neural progenitor cells were loaded with magnetic nanoparticles and delivered to mice with melanoma. Using an alternating magnetic field, hyperthermia was induced and significant tumor attenuation was observed [126]. A similar strategy might prove effective for glioma targeting. Results thus far combining stem cells and nanoparticles for the induction of toxicity towards glioma cells demonstrate a simple and promising proof-of-concept, but more work needs to be conducted in order to show that stem cells improve the efficacy of free-standing nanoparticle systems.

### 6.4 Modulation of stem cell behavior

The behavior and characteristics of stem cells may be altered by loaded nanoparticles, offering new avenues for therapy. Chung et al. in a recent report demonstrated that MSCs loaded with iron oxide nanoparticles showed increased EGFR expression [127]. This resulted in more efficient MSC migration towards tumor cells. It also allowed MSCs to interfere with tumor EGF/EGFR growth signaling, tumor angiogenesis, and VEGF expression. Although this was demonstrated in the context of colon cancer, such strategies could be used to target malignant gliomas as EGFR has been shown to be amplified in glioblastoma multiforme [128]. It has also been demonstrated that ferucarbotran-protamine complex-labeled hMSCs had a higher expression of CXCR4, a receptor that is thought to be critical for stem cell migration towards glioma cells [129]. Such modifications to stem cells could improve migration efficiency and offer unique ways of targeting malignant gliomas.

## 7 Challenges and potential pitfalls of nanoparticle-loaded stem cells in anti-glioma therapy

The use of nanoparticles may offer improvements for glioma therapy on several fronts. They can bring the added benefit of enhancing tumor imaging while simultaneously causing toxicity to glioma cells. Stem cells could provide the effective targeted delivery mechanism that is currently lacking in nanomedicine. However, there are challenges that must be overcome in order to develop effective therapeutic systems using such a combination. Highlighted are some limitations that need to be addressed (Table 1).

**Table 1:** Attractive features and potential pitfalls of drug-loaded nanoparticle systems incorporated in stem cell carriers

Attractive Features	Potential Pitfalls
Sustained drug release to tumor cells	Low intratumoral distribution
Targeted incorporation in neoplastic cells	Drugs must be stably loaded into the stem cell carrier
Accepts incorporation of potent chemotherapeutic agents	Allorejection of heterologous stem cell carriers
High loading capacity per nanocarrier	Non-representative animal models
Oncotropic properties	Difficulties in controlling off-target toxicity
Able to reach invasive tumor areas	Difficulty in achieving controlled drug release

### 7.1 Toxicological issues

Every year bigger investments have been directed to the development of new nanoparticle delivery systems for anti-cancer therapy. Nevertheless, the possible effects of these nanocarriers on the tumor microenvironment and on the host biology have not yet been fully clarified. Recent data have suggested that the physiochemical properties of nanoparticles may lead to unpredictable biological interactions and undesirable toxicity. Pan Y et al. have demonstrated the existence of size-dependent nanoparticle cytotoxicity. In this study, small sized gold nanoparticles (1.4 nm) were shown to induce programmed cell death by intercalating into the DNA major groove. However, larger gold nanocarriers (more than 15 nm) demonstrated no toxicity [130]. Another report has revealed the propensity of single-walled carbon nanotubes to disrupt the nuclear mitotic spindle apparatus and thus interfere with cell division [131]. The relative surface area and the coating of a nanocarrier have also been correlated with cytotoxic effects. Monteiller et al. recently determined the role of surface area on the pro-inflammatory effects of low-toxicity low-solubility nanoplatforms. They have shown both *in vivo* and *in vitro* that the high surface area of a nanoparticle was a key factor in their inflammogenicity [132]. Similarly, Valhov H et al. and Kagen VE et al., respectively, showed that endotoxin adherence on a nanoparticle's surface as well as transition metal contamination during its production could result in unwanted adverse reactions [133, 134]. Nanocarrier-related toxicity can also occur due to ROS (reactive oxygen species) generation. Many mechanisms have been proposed to explain such an event. Amongst them, the presence of metal contaminants, particle dissolution after internalization in the cell and membrane damage by bigger nanoplatforms have been directly related with ROS production and cell death [135]. However, toxicity caused by nanoparticles has not only been correlated with off-target effects. New cancer immunotherapies have been taking advantage of immunogenic nanomaterials to induce host vaccination [136]. Therefore, nanocarriers can be used to both deliver the therapeutic agent and activate the immune system against the tumor.

### 7.2 Evaluation of efficacy and safety

The efficacy of a nanoparticle-delivery system can be affected by its shape, size, stability, density, solubility, and surface charge. Therapeutic efficacy has also shown to be directly correlated with the drug-release profile. Consequently, the mechanism that triggers drug release may influence the bioavailability and biodistribution of the therapeutic agent [137, 138]. A couple of clinical trials have tested both efficacy and safety of some specific nanomedicines in anti-cancer therapy. They have demonstrated that nanoparticle uptake also depends on the tumor microenvironment and vascularization, which facilitates the uptake of drug-loaded nanocarriers. A phase III clinical trial compared the efficacy of pegylated-liposomal doxorubicin versus the combination of doxorubicin, bleomycin and vincristine in 258 patients with advanced Kaposi's sarcoma, a highly vascularized tumor. A clear-cut improvement in therapeutic response, overall survival and decreased off-site toxicity was observed in the group treated with pegylated-liposomal doxorubicin [139]. On the other hand, a phase III trial evaluating the efficacy and safety of pegylated-liposomal doxorubicin versus doxorubicin alone in patients with metastatic breast cancer showed decreased cardiotoxicity, but was unable to show an increased therapeutic effect of drug-loaded nanoparticles compared to free doxorubicin [140]. The above results indicate that multiple factors influence both efficacy and safety of drug-loaded nanocarriers. Therefore, future studies should focus on the development of more biocompatible nanoplatforms that would be able to facilitate combined therapeutic regimens in order to target different disease pathways, surpass difficulties imposed by tumor biology and microenvironment and increase both safety and therapeutic efficacy.

### 7.3 Migration efficiency of nanoparticle-loaded cell carriers towards neoplastic tissues

The loading efficiency of nanocarriers into stem cells can be low. Therefore, in order to be able to deliver a therapeutically effective drug dose, a sufficient number of cells must migrate to the targeted tumor area. Although stem cells have demonstrated an ability to migrate towards glioma cells, the migration efficiency is still considered low [78]. To overcome this issue, methods need to be developed to increase the pool of migrating stem cells. Klopp et al. demonstrated that tumor irradiation increased the recruitment of circulating MSCs into the tumor microenvironment [141]. Pre-treatment with irradiation may thus offer a way to increase the number of migrating cells in a future therapy. Growth factor injection or chemokine co-injection may also activate certain stem cells to become more migratory. Kendall et al. demonstrated that several signaling molecules produced by glioma cells activate the phosphatidylinositide 3-kinase (PI3K) pathway [142]. Stimulation of this pathway may enhance the sensitivity of these cells towards signals produced by the tumor. The type of stem cell used may also influence the migration efficiency of nanoparticle-loaded cell carriers. NSCs may be more suited for delivery of nanoparticles to glioma when compared to other stem cell types. Ahmed et al. found that NSCs loaded with an oncolytic adenovirus displayed more tumor-specific migration when compared to MSCs carrying the same virus [11]. A further understanding of the signaling pathways mediating stem cell migration may offer a means of increasing the migrating pool of stem cells in the future.

### 7.4 Maintaining optimal drug stability and release

To be effective, a therapeutic agent must first be stably loaded inside the cell carrier and then be released into the extracellular space. Rather than just a quick drug release, a slow and steady discharge is necessary in order to reach as many tumor cells as possible. Both drug stability and controlled release are dependent on the mechanism of intracellular trafficking utilized by the nanoparticle. Drug stability can be achieved by avoiding the entry of loaded nanoplatforms into lysosomes [143] or by rapid endo-lysosomal escape [106, 107]. A recent study demonstrated that silver nanoparticles were internalized by human mesenchymal stem cells (hMSCs) via a clathrin-dependent mechanism. Once inside the cells, stable silver agglomerates could be observed in the perinuclear region [144]. An additional report showed that ritonavir nanoparticles coated with poloxamer 188 and assembled by high-pressure homogenization were internalized by human monocyte-derived macrophages through a clathrin-dependent mechanism. These nanoplatforms remained stable inside endosomes and were then discharged by Rab11 (Ras-related protein) and Rab14-dependent mechanisms. The released nanoparticles kept their ability to preclude HIV-1 infection in human monocyte-derived macrophages [95].

Delayed drug release is also necessary to give the cell carrier time to migrate towards the tumor. This can be accomplished via “internal” and “external” triggers. Some nanoparticles have been designed with a pH-sensitive linker that releases drugs or peptides upon nanoparticle entry into the lysosome [145]. Fang et al. developed a nanoparticle system containing SPIO nanoparticles, a pH-sensitive poly (beta-amino ester) (PBAE) copolymer, and doxorubicin. This particle demonstrated drug release at pH 5.5 and 6.4 corresponding to the endosome [146]. Other intrinsic release mechanisms include glutathione-mediated controlled release where disulfide linkers are broken at tumor-relevant glutathione levels [147]. However, for stem cell delivery of such nanoparticles, it may be more beneficial to have an external stimuli-mediated toxicity to ensure that stem cell migration can be achieved. Some external triggers that have been used to allow for release of attached molecules from nanoparticles include ultrasound release of drugs [148], photothermal linkers that break in response to heat, and photo-mediated release [149]. In addition to this, other methods such as photothermal ablation allow for control of a damaging stimulus to tumor cells [150]. Although highly promising, the study of the mechanisms behind drug stability and release from stem cell carriers is still in its infancy. Additional efforts should be invested to uncover new pathways and thus advance this research field.

### 7.5 Translational issues

An important pitfall regarding the implementation of nanoparticle-loaded stem cell carriers into the clinical setting for targeted anti-cancer therapy is the current use of non-representative animal models in pre-clinical studies. These models are not able to fully exemplify the heterogeneous and complex nature of high-grade gliomas, since rodent tumors grow much faster than human (weeks vs years) and therefore they contain a much higher vascular permeability. As more nanoparticle will extravasate leaky vessels, the sole use of rapidly growing rodent models may lead to an overestimation of the potential usefulness of passively targeted drug-loaded nanoparticle systems [151]. Another critical point is that most preclinical studies are performed in immunocompromised mouse models, which exclude the possibility of evaluating the involvement of the host-immune system on the therapeutic outcome. Last, when compared to stem cell carriers transplanted to human patients, preclinical rodent models are not representative of the actual migration distance that these carriers need to achieve in order to reach invasive tumor areas. Due to the discrepancies between preclinical and clinical models, it can be argued that many agents that are effective in animal models may not fulfill expectations in humans.

Another fundamental point that needs to be addressed when considering the use of stem cell carriers in the clinical setting is the origin of these cells. Heterologous stem cell carriers can be subject to HLA mismatches and, consequently, host-related rejection [152, 153]. Cell carriers that are derived from the own patient (autologous origin) are not subject to alloimmunization. However, their isolation from human patients and expansion in culture is so difficult, time-consuming, and expensive that, unless alternative methods are developed, the clinical implementation of this technique becomes almost impossible [154, 155].

## 8 CONCLUSION AND FUTURE PERSPECTIVES

The design of new nanoparticle systems for the treatment of malignant gliomas has the objective of developing both safer and more effective diagnostic and therapeutic approaches. In order to fulfill their full potential and serve as candidates for targeted drug delivery, nanoparticles must present a versatile structure, which should be amenable to small molecules, peptides, and a wide variety of chemotherapeutic agents. Their construction should be biodegradable, non-toxic and biocompatible, so they could deliver their therapeutic payload anywhere in the brain without unwanted adverse reactions due to nanoparticle accumulation or activation of a host-related inflammatory response. They should also present a controlled drug release profile, which would secure continuous drug delivery at the targeted neoplastic tissue. Upon reaching the malignant area, they should first avoid the mononuclear phagocyte system and then be effectively recognized and internalized by glioma cells. This would allow for a direct and efficient payload distribution.

Recent preclinical and clinical studies have repeatedly underscored the importance of drug-loaded nanoparticles in anti-cancer diagnosis and therapy. Nevertheless, the use of drug-encapsulated nanomaterials alone as a therapeutic tool still poses some important limitations that hamper complete neoplastic destruction. Two important examples are uneven intratumoral distribution and inefficient targeting of disseminated tumor areas. The combination of nanoparticle delivery systems with stem cell carriers could potentially overcome these limitations, increasing both the efficiency and efficacy of nanotherapy. Due to their inherent tumor-tropic and immunosuppressive properties, stem cells carriers can not only serve as optimal vehicles for distant delivery, but can also manipulate the tumor microenvironment in order to assist in the eradication of tumor cells. Moreover, these cell carriers can be loaded with multifunctional drug-loaded radiolabeled nanomaterials. This combination could be used as an extra instrument to report *in vivo* efficacy of the therapeutic agent and to track the fate of stem cell carriers.

Although a promising strategy, the use of stem cell carriers combined with drug-loaded nanoparticles for anti-glioma therapy still faces some important challenges. First, there should be a better characterization of molecular targets that are specific to malignant gliomas, and are not present in non-neoplastic tissues. As a consequence, drug-loaded nanomaterials could be functionalized for a highly targeted approach. Second, as nanoparticle-delivery systems support a high drug loading per carrier, more therapeutic agents are able to reach the targeted area. To avoid unwanted toxicity, recalibration of drug dosage may be necessary. Third, regarding the manufacturing process, these nanovehicles should have their formulation design optimized in order to increase their reproducibility, cost-effectiveness and biocompatibility. This would open a new path for redirecting nanomedicine formulations towards industrial utilization and clinical translation. Last, more efforts should be placed on a further characterization of stem cell carriers and on the determination of their biological function in the tumor microenvironment. This would allow for a more reliable therapeutic exploitation of these cell carriers.

More investments should be directed to the development of multifunctional nanoplatforms, which consist of both drugs and imaging agents within the same nanoparticle delivery system. Such an approach would allow for effective tracking of the destination and biodistribution of cell carriers, nanoparticles and therapeutic agents, permitting a non-invasive real-time visualization of the efficacy of the intervention. This information could actively influence the decision to adjust drug dosage or to whether or not continue the therapy. Image-guided insights would also assist in the pre-screening of patients based on the profile of nanoparticle assimilation by the tumor: active or passive targeting [156-158]. Such knowledge might contribute to the development of personalized medicine, where the best therapeutic response for each patient would be predicted. Future efforts should be directed towards better understanding the biological and pathophysiological principles of stem cell delivery to neoplastic areas, nanoparticle loading of stem cells, biodistribution and assimilation of nanomedicines by tumor cells, and drug targeting. It would allow us to obtain a more in-depth understanding into the shortcomings of stem cell-based tumor-targeted drug delivery, and would assist us in finding optimal strategies to surpass these limitations in the future.
